# Impact of Sugammadex on Clinical and Patient-Centered Outcomes: Beyond Neuromuscular Recovery

**DOI:** 10.3390/jcm15176670

**Published:** 2026-08-28

**Authors:** Ji-Yoon Jung, Tae-Yun Sung

**Affiliations:** Department of Anesthesiology and Pain Medicine, Konyang University Hospital, Konyang University Myunggok Medical Research Institute, Konyang University College of Medicine, 158 Gwanjeodong-ro, Seo-gu, Daejeon 35365, Republic of Korea; 200756@kyuh.ac.kr

**Keywords:** sugammadex, anesthesia, general, neuromuscular blockade, postoperative complications, treatment outcome, cholinergic antagonists

## Abstract

**Background**: Residual neuromuscular blockade remains an important contributor to postoperative morbidity. Sugammadex provides rapid and predictable reversal of rocuronium- and vecuronium-induced blockade and reduces residual neuromuscular blockade more effectively than conventional anticholinesterase-based reversal. **Methods**: This structured narrative review was informed by searches of PubMed/MEDLINE and the Cochrane Library from database inception to 20 August 2026. Reference lists of relevant guidelines, systematic reviews, and eligible studies were also screened. Randomized trials, observational studies, systematic reviews, and meta-analyses reporting clinical or patient-centered outcomes after sugammadex or neostigmine-based reversal were prioritized. **Results**: The most consistent evidence relates to neuromuscular recovery and residual blockade. Observational studies and meta-analyses suggest possible reductions in selected postoperative pulmonary complications with sugammadex, but findings are heterogeneous and do not establish a uniform causal benefit. Potential differences have also been reported in hemodynamic events, urinary retention, early postoperative nausea and vomiting, and early physiological recovery. Evidence regarding postoperative pain, delirium, overall morbidity, mortality, length of stay, healthcare costs, and patient-reported recovery remains mixed and context-dependent. Important safety considerations include bradycardia, hypersensitivity and anaphylaxis, transient changes in coagulation assays, recurrent block after inadequate dosing, and prolonged exposure in severe renal impairment. **Conclusions**: Sugammadex may be associated with favorable selected postoperative outcomes, but it should not be regarded as an independent determinant of recovery. Its clinical effects must be interpreted as part of optimized neuromuscular management incorporating quantitative monitoring, blockade-depth-appropriate dosing, confirmation of recovery, and comprehensive perioperative care.

## 1. Introduction

Neuromuscular blockade is a commonly used component of general anesthesia that facilitates endotracheal intubation and mechanically controlled ventilation by providing muscle relaxation and contributes to creating optimal surgical conditions and reducing the requirement for anesthetics [1].

Conventional reversal of neuromuscular blockade using anticholinesterase–anticholinergic combinations has traditionally focused on restoring neuromuscular transmission, typically assessed by train-of-four (TOF) recovery. However, this strategy is limited by its indirect mechanism of action, substantial inter-individual variability, and a ceiling effect, particularly in the setting of deep neuromuscular blockade [2]. Consequently, incomplete recovery or residual neuromuscular blockade (RNB) remains common in clinical practice and is associated with clinically significant postoperative morbidity, particularly respiratory compromise and airway vulnerability. In addition, the use of conventional reversal is frequently accompanied by undesirable muscarinic and anticholinergic adverse effects.

Sugammadex, a selective relaxant binding agent, represents a paradigm shift in the reversal of aminosteroidal neuromuscular blocking agents by directly encapsulating free molecules of rocuronium and vecuronium, thereby enabling rapid, predictable, and complete reversal, even from deep levels of blockade when the appropriate dosage is administered [3]. While its efficacy in achieving reliable neuromuscular recovery is well established, growing attention has been directed toward its potential to influence a broader spectrum of perioperative outcomes.

Emerging evidence has examined whether sugammadex use is associated with outcomes beyond the prevention of RNB, including postoperative pulmonary complications (PPCs), hemodynamic events, postoperative nausea and vomiting (PONV), postoperative urinary retention (POUR), postoperative pain, postoperative delirium (POD), mortality, and quality of recovery. However, these outcomes are influenced by multiple perioperative factors, and much of the available evidence is observational. Apparent benefits attributed to sugammadex may therefore reflect, at least in part, broader optimization of neuromuscular management, including quantitative monitoring, selection and dosing of the reversal agent according to blockade depth, and confirmation of adequate recovery before extubation.

In this context, this review aims to critically evaluate clinical and patient-centered outcomes associated with sugammadex beyond neuromuscular recovery. Particular attention is given to study design, consistency of findings, plausible mechanisms, safety, and the distinction between the pharmacologic effect of sugammadex and the broader effect of optimized neuromuscular management.

## 2. Methods

This structured narrative review was informed by a literature search of PubMed/MEDLINE and the Cochrane Library from database inception to 20 August 2026. The search combined terms related to the intervention and comparator (“sugammadex,” “neostigmine,” “neuromuscular blockade reversal,” “rocuronium,” and “vecuronium”) with terms related to outcomes and safety (“residual neuromuscular blockade,” “postoperative pulmonary complications,” “pneumonia,” “respiratory failure,” “hemodynamic,” “bradycardia,” “postoperative nausea and vomiting,” “urinary retention,” “catheter-related bladder discomfort,” “pain,” “delirium,” “quality of recovery,” “length of stay,” “mortality,” “anaphylaxis,” “hypersensitivity,” “coagulation,” “bleeding,” “recurrent block,” and “renal impairment”). Reference lists of relevant practice guidelines, systematic reviews, meta-analyses, and eligible primary studies were screened to identify additional publications.

English-language randomized controlled trials, prospective and retrospective observational studies, systematic reviews, meta-analyses, and major clinical guidelines reporting clinical or patient-centered outcomes after sugammadex or neostigmine-based reversal were considered. For common outcomes, higher-level evidence and large contemporary comparative studies were prioritized; case reports and pharmacovigilance reports were considered primarily for rare but clinically important safety events. One author (JY-J) performed the search and initial selection, and both authors reviewed the included literature and agreed on its interpretation. Because the review addressed multiple outcomes with substantial heterogeneity in populations, procedures, comparator regimens, outcome definitions, assessment times, and study designs, a pooled quantitative synthesis across outcomes was considered clinically inappropriate. Findings were therefore synthesized narratively, with randomized and observational evidence explicitly distinguished and causal language limited according to study design.

## 3. Pharmacologic and Mechanistic Differences

The pharmacologic and mechanistic differences between sugammadex and conventional anticholinesterase-based reversal agents underpin their distinct clinical profiles and may contribute to differences in perioperative outcomes (Table 1). Whereas anticholinesterases such as neostigmine act indirectly by inhibiting acetylcholinesterase at the neuromuscular junction, sugammadex selectively encapsulates aminosteroidal neuromuscular blocking agents (NMBAs), primarily rocuronium and vecuronium [4]. This binding lowers the free plasma concentration of aminosteroidal NMBAs and creates a concentration gradient that promotes dissociation of the NMBA from nicotinic receptors [5]. Sugammadex can reverse moderate, deep, and—when clinically necessary—immediate rocuronium-induced blockade, but efficacy and the required dose depend on the measured depth of blockade. Avoidance of cholinergic modulation also removes the need for routine anticholinergic coadministration, although sugammadex has its own important adverse-effect profile.

In clinical practice, these mechanistic differences have several implications. First, sugammadex reversal is dose dependent and must be matched to the measured depth of neuromuscular blockade. When an appropriate dose is selected using quantitative monitoring, reversal is generally rapid and predictable across a wider range of blockade depths than is possible with neostigmine [6]. In contrast, anticholinesterase efficacy is constrained by a ceiling effect and depends strongly on spontaneous recovery at the time of administration. Incomplete or delayed reversal is therefore more likely when neostigmine is given during deeper blockade. The maximum useful effect of neostigmine generally occurs at approximately 0.04–0.05 mg/kg; larger doses do not overcome profound blockade and may impair upper-airway dilator function [7,8]. Current guidelines recommend sugammadex over neostigmine for rocuronium-induced deep, moderate, and shallow blockade and consider neostigmine a reasonable alternative primarily when recovery has reached a minimal depth and quantitative monitoring is available [2,9].

Second, the ability of appropriately dosed sugammadex to reverse deep neuromuscular blockade has implications for intraoperative management. Deeper blockade may improve surgical conditions in selected laparoscopic and spinal procedures [10,11], but its clinical use was historically constrained by concern about delayed or incomplete reversal. After the introduction of sugammadex, one retrospective cohort documented a 45.1% increase in intraoperative rocuronium dosing [12]. This change illustrates that postoperative outcomes attributed to sugammadex cannot always be separated from changes in intraoperative NMBA exposure, blockade depth, monitoring, and surgical conditions. Whether deeper blockade improves patient-centered outcomes remains procedure specific and uncertain [11].

Finally, sugammadex does not rely on modulation of acetylcholine levels and therefore avoids the activation of muscarinic receptors that necessitates the co-administration of anticholinergic agents when using neostigmine. The anticholinesterase–anticholinergic combination is associated with a range of autonomic side effects. Muscarinic side-effects caused by anticholinesterase include prolongation of the QT interval of the electrocardiograph (ECG), bradycardia, PONV, propulsive bowel activity, miosis, bronchoconstriction, and ptyalism [4]. Side effects of anticholinergics administered to prevent muscarinic side effects include tachycardia, urinary retention, blurry vision, and decreased intestinal motility [13]. On the other hand, sugammadex is not associated with QT interval prolongation and can reverse neuromuscular blockade without autonomic effects [6,14], providing a physiologically more effective reversal approach and potentially having a better side effect profile.

## 4. Impact of Sugammadex and Neuromuscular Monitoring on Neuromuscular Recovery

The most well-established advantage of sugammadex over neostigmine-based reversal is more rapid and predictable recovery when the dose is selected according to the depth of rocuronium- or vecuronium-induced blockade. Multiple meta-analyses have shown shorter times to a TOF ratio ≥ 0.9, shorter extubation times, and a lower incidence of postoperative RNB with sugammadex [15,16,17,18]. These pharmacodynamic advantages may support operating-room efficiency and early postoperative safety, but their magnitude depends on blockade depth, dosing, monitoring technique, and workflow.

Nevertheless, sugammadex does not replace quantitative neuromuscular monitoring. Without quantitative monitoring, clinicians cannot reliably determine blockade depth, select the appropriate dose, or exclude RNB even after a recommended dose [19,20]. In a retrospective cohort, the association between higher rocuronium dose and respiratory complications was no longer observed when quantitative rather than qualitative monitoring was used [12]. This finding should not be interpreted as complete elimination of PPCs; rather, it suggests that quantitative monitoring may mitigate one pathway linking NMBA exposure to respiratory complications. Thus, quantitative monitoring and confirmation of recovery remain essential regardless of the reversal agent.

To ensure neuromuscular recovery, a TOF ratio of ≥0.9 is required when using electromyography (EMG). In the case of acceleromyography (AMG), measurements should ideally be normalized to the baseline value, as unnormalized ratios often overestimate recovery. When using non-normalized AMG, a more stringent threshold of ≥1.0 is recommended [9].

## 5. Impact on Perioperative Outcomes

The clinical implications of neuromuscular blockade reversal extend beyond the restoration of neuromuscular transmission to encompass a wide spectrum of perioperative outcomes. By minimizing RNB and avoiding cholinergic side effects, sugammadex may influence multiple organ systems and recovery domains during the perioperative period. A summary of the directions, proposed mechanisms, strength of evidence, and key references for each outcome is provided in Table 2.

### 5.1. Respiratory Outcomes

Among perioperative outcomes, respiratory complications remain the most directly linked to the adequacy of neuromuscular recovery. NMBAs dose-dependently contribute to RNB and postoperative respiratory morbidity [8,12,21].

RNB impairs ventilatory function, hypoxic ventilatory response, and upper airway integrity; even subtle incomplete recovery can impair pharyngeal coordination and airway protection, increasing the risk of upper airway obstruction, hypoxemia, aspiration, atelectasis, and pneumonia [22,23,24]. Large observational studies, including the POPULAR study, have also linked NMBA exposure and cumulative NMBA dose with an increased incidence of PPCs [8,21].

Sugammadex may reduce selected postoperative respiratory events by decreasing RNB more reliably than neostigmine, but direct evidence for downstream pulmonary benefit is less consistent than evidence for neuromuscular recovery. In the STRONGER multicenter matched cohort study of 45,712 adults, sugammadex use was associated with lower rates of major PPCs, pneumonia, and respiratory failure than neostigmine use [25]. Because this was an observational matched-cohort analysis, residual confounding and differences in monitoring and perioperative practice cannot be excluded. Meta-analyses combining randomized and observational evidence have reported lower risks of selected events, including pneumonia, atelectasis, noninvasive ventilation, reintubation, and respiratory failure, whereas effects on transient desaturation, length of stay, patient-reported recovery, and mortality have been inconsistent [26,27,28].

However, the respiratory benefit of sugammadex remains uncertain in some settings. A large multicenter retrospective cohort study involving 83,250 patients found no significant difference between sugammadex and neostigmine in a composite respiratory outcome including post-extubation desaturation, respiratory failure requiring noninvasive ventilation, or reintubation [29]. More recent registry-based data also reported a statistically higher incidence of PPCs with sugammadex than with neostigmine, although the absolute difference was small and may not be clinically meaningful [30]. These discrepancies suggest that the effect of sugammadex on PPCs is modified by patient risk, surgical type, NMBA dosing, depth of block at reversal, monitoring practices, and perioperative respiratory care.

Overall, high-level evidence supports a reduction in RNB with appropriately dosed sugammadex, whereas evidence for prevention of clinically defined PPCs remains heterogeneous. Current guidelines emphasize quantitative monitoring, confirmation of a TOF ratio ≥ 0.9 before extubation, and selection of the reversal strategy according to blockade depth [2]. Until adequately powered randomized trials clarify patient-centered respiratory outcomes, sugammadex should be regarded as one component of a broader respiratory-risk-reduction strategy rather than a stand-alone protective intervention [31].

### 5.2. Hemodynamic Outcomes

Bradycardia is a well-recognized potential adverse effect of sugammadex. Early pharmacovigilance data from the U.S. Food and Drug Administration Adverse Event Reporting System (FAERS) between 2009 and 2017 suggested a higher number of reported cardiac adverse events with sugammadex compared with neostigmine. However, these findings should be interpreted with caution due to the inherent limitations of spontaneous reporting systems, including the absence of a denominator (i.e., total exposed population) and the potential for reporting bias following the introduction of a new drug [32].

In contrast, comparative clinical evidence does not demonstrate a consistent increase in cardiovascular complications with sugammadex. A large propensity-matched cohort found no significant difference in major adverse cardiovascular events between sugammadex and neostigmine [33]. Randomized trials [34,35] and meta-analyses [18,36] have reported comparable or lower rates of bradycardia and other hemodynamic disturbances with sugammadex than with neostigmine-based reversal. These comparative findings should not obscure the rare possibility of profound bradycardia or cardiac arrest shortly after sugammadex administration; continuous monitoring and readiness to treat clinically important bradycardia remain necessary [32].

### 5.3. Postoperative Nausea and Vomiting (PONV)

Neostigmine may increase the risk of PONV via cholinergic mechanisms, but this effect is likely attenuated by concomitant anticholinergic agents, particularly atropine, which may exert central antiemetic effects [37]; evidence-based analyses suggest that routine doses do not significantly increase PONV risk, although higher doses (≥2.5 mg) may be associated with an increased incidence [38].

Randomized and observational studies comparing sugammadex with neostigmine-based reversal have produced heterogeneous PONV findings. Some trials and pooled analyses suggest a reduction, particularly during the early postoperative period [37,39,40,41], whereas other systematic reviews and trials show no significant difference [15,42]. Variation in baseline PONV risk, surgical procedure, neostigmine and anticholinergic dose, timing of assessment, and antiemetic prophylaxis limits causal interpretation. Sugammadex should therefore not be regarded as an antiemetic intervention, and reversal choice should not replace guideline-based multimodal PONV prophylaxis.

Notably, studies reporting a possible benefit of sugammadex have generally identified differences during the early postoperative period: at arrival in the post-anesthesia care unit (PACU) after extremity surgery [39], at 1 h after elective surgery [40], or within 6 h after ear–nose–throat procedures [37]. These time-limited findings further support cautious interpretation and do not demonstrate a sustained effect on overall PONV burden.

### 5.4. Genitourinary Outcomes

Genitourinary symptoms such as postoperative urinary retention (POUR) and catheter-related bladder discomfort (CRBD) represent underrecognized but clinically relevant contributors to postoperative morbidity and patient dissatisfaction.

Bladder contraction is primarily mediated by muscarinic receptors, particularly the M3 subtype receptors [43]. Anticholinergic agents such as atropine and glycopyrrolate, which are routinely co-administered with neostigmine, act as nonselective muscarinic antagonists that reduce bladder detrusor muscle contractility, potentially increasing the risk of POUR but reducing CRBD [44,45]. In contrast, sugammadex does not interact with muscarinic receptors and therefore lacks antimuscarinic effects, providing a biologically plausible basis for differences in urinary outcomes. This mechanistic distinction is supported by recent large-scale evidence. A meta-analysis by Ni et al., including 25 studies and over 160,000 patients, demonstrated a significantly lower incidence of POUR with sugammadex compared with neostigmine or no reversal (relative risk 0.47, 95% confidence interval 0.34–0.64; *p* < 0.001), suggesting a clinically meaningful reduction in urinary complications [44]. However, it is important to note that prospective studies specifically designed with POUR as a primary endpoint remain lacking, and most available data are derived from observational analyses or secondary outcomes, which may limit the strength of causal inference.

In contrast, CRBD, characterized by a burning sensation, urgency to void, or suprapubic discomfort, appears to be modulated in the opposite direction. Because CRBD is mediated by muscarinic receptor activation and involuntary bladder contractions, anticholinergic agents may alleviate these symptoms. Consistent with this mechanism, a prospective randomized controlled trial in patients undergoing transurethral resection of bladder tumors demonstrated that neostigmine combined with atropine significantly reduced both the incidence and severity of CRBD at 0, 1, 6, 12, and 24 h postoperatively compared with sugammadex [46]. Furthermore, glycopyrrolate appears to provide greater reduction in CRBD than atropine, potentially due to its higher affinity for muscarinic M3 receptors [45].

Thus, the genitourinary effects of reversal strategy appear bidirectional: sugammadex may reduce POUR by avoiding antimuscarinic inhibition of detrusor contractility, whereas anticholinergic coadministration with neostigmine may reduce CRBD in catheterized patients.

### 5.5. Postoperative Pain

Evidence from randomized controlled trials suggests that any observed differences in pain may depend on surgical context and associated physiological factors. In a study of morbidly obese patients undergoing laparoscopic bariatric surgery, sugammadex was associated with lower visual analogue scale (VAS) pain scores at 30 and 60 min after arrival in the PACU compared with neostigmine-based reversal [47]. This effect has been attributed by the authors to a combination of factors, including increased gastrointestinal motility with neostigmine, smoother emergence with sugammadex, and a lower incidence of postoperative nausea and vomiting in the sugammadex group, all of which may influence early postoperative comfort.

In contrast, another prospective randomized study in patients undergoing transurethral resection of bladder tumors reported lower postoperative numerical rating scale (NRS) pain scores in the neostigmine–atropine group at all assessed time points (0, 1, 6, 12, and 24 h postoperatively) [46]. The authors suggested that this finding may reflect reduced catheter-related bladder discomfort due to the antimuscarinic effects of atropine, with the possibility that patients may perceive or report bladder discomfort as surgical pain, thereby lowering apparent pain scores in this group.

Overall, the impact of neuromuscular blockade reversal on postoperative pain appears to be limited and largely indirect. Sugammadex does not possess intrinsic analgesic properties, and reported findings on postoperative pain are inconsistent and likely reflect indirect, context-specific effects rather than true analgesic differences. Accordingly, current evidence suggests that the choice of reversal agent has minimal direct impact on postoperative pain, and pain management should remain grounded in established multimodal analgesic strategies.

### 5.6. Postoperative Delirium (POD)

Postoperative delirium (POD) is a multifactorial condition, with central cholinergic deficiency considered a key contributing mechanism. Neostigmine increases synaptic acetylcholine levels and may theoretically support central cholinergic activity; however, its clinical effects are modulated by the concomitant use of anticholinergic agents. Among these, atropine readily crosses the blood–brain barrier and may increase central anticholinergic burden, potentially impairing cholinergic signaling, whereas glycopyrrolate has limited central penetration and is therefore less likely to exert such effects [48]. In addition, RNB may contribute to hypoxia and pulmonary complications, further increasing the risk of POD [49].

Sugammadex may influence delirium risk indirectly by providing more reliable reversal of neuromuscular blockade, reducing RNB and related respiratory compromise, and avoiding routine anticholinergic coadministration [17,23,50]. However, these mechanisms remain inferential, and the clinical relationship between reversal strategy and POD has not been fully established [51].

A recent systematic review and meta-analysis included seven randomized controlled trials (RCTs; *n* = 759) and five non-randomized studies (*n* = 50,115) [51]. A lower incidence of POD within 24 h was observed in the pooled RCTs but not in the non-randomized studies. The RCTs were small, often excluded patients at high risk for delirium, and used heterogeneous assessment methods. Subgroup analyses showed no clear difference according to the anticholinergic administered with neostigmine and no consistent difference in perioperative Mini-Mental State Examination scores. These findings support a possible early association but do not establish that sugammadex prevents delirium.

Overall, the available evidence supports a potential early reduction in POD with sugammadex, but further well-designed RCTs using standardized assessment methods are needed.

### 5.7. Overall Postoperative Morbidity and Mortality

Beyond respiratory outcomes, differences in reversal strategy may be associated with broader postoperative morbidity through the adequacy of neuromuscular recovery and avoidance of cholinergic-anticholinergic effects. However, evidence for global outcome improvement is inconsistent and should not be attributed to sugammadex alone. A systematic review and meta-analysis found lower rates of some PPCs but no consistent improvement in hospital length of stay, patient-reported quality of recovery, or mortality [28].

Observational studies have reported associations with selected longer-term outcomes, including lower 90-day mortality in some cohorts [52], but these findings remain vulnerable to confounding by patient selection, institutional practice, monitoring, and perioperative care. In patients with end-stage renal disease, a single-center propensity score-matched study found no significant difference in 30-day or 1-year mortality according to sugammadex exposure [53]. Mortality is a distal, multifactorial endpoint; current evidence does not support a causal survival benefit from sugammadex.

### 5.8. Postoperative Recovery and Quality of Recovery (QoR)

A randomized controlled trial using the Postoperative Quality Recovery Scale suggests that sugammadex may improve early physiological recovery without consistently affecting broader recovery domains. Assessments were performed preoperatively and at 15 and 40 min and 1 day postoperatively; however, sugammadex was associated with a higher recovery rate only in the physiological domain at 15 min, with no significant differences at other time points or in other QoR domains [54].

In pediatric cardiac surgery, sugammadex was associated with shorter extubation time and fewer pulmonary complications, which may facilitate recovery in high-risk populations [55]. Observational data further suggest system-level benefits. In a large multicenter registry study of adult patients undergoing ambulatory surgery, sugammadex use was associated with a reduction in postoperative length of stay in the ambulatory care facility and healthcare costs compared with neostigmine-based reversal, partly mediated by a lower incidence of postoperative nausea and vomiting [56].

However, evidence from systematic reviews and meta-analyses indicates that these advantages do not consistently translate into improvements in overall quality of recovery. While sugammadex reduces RNB and postoperative complications, pooled analyses have shown no significant differences in hospital length of stay, patient-reported QoR, or postoperative cognitive outcomes compared with neostigmine-based reversal [28]. Overall, the impact of reversal strategy on QoR appears to be domain-specific and time-dependent, and improvements in physiological recovery do not necessarily translate into enhanced global quality of recovery.

**Table 2:** Summary of evidence on clinical and patient-centered outcomes associated with sugammadex compared with neostigmine-based reversal.

Outcome	Directions of Evidence	Main Proposed Mechanism	Evidence Base	Key References
RNB	Consistently favors sugammadex	Rapid and complete reversal	RCTs and meta-analyses; consistent	[2,9,15,16,17,18,19,20]
PPCs	Generally, favors sugammadex, but inconsistent	Less RNB and improved airway protection	Predominantly observational; meta-analyses heterogeneous	[8,12,21,25,26,27,28,29,30,31]
Hemodynamic events	Comparable or favors sugammadex	Avoidance of cholinergic–anticholinergic effects	RCTs, observational studies, and meta-analyses; mixed	[18,32,33,34,35,36]
PONV	Mixed; possible early benefit	Avoidance of cholinergic stimulation	Small RCTs and meta-analyses; mixed	[5,37,38,39,40,41,42]
POUR	Favors sugammadex	Avoidance of antimuscarinic bladder inhibition	Predominantly observational and secondary outcomes	[43,44,45]
CRBD	May favor neostigmine-based reversal	Antimuscarinic bladder effect	Limited RCT evidence	[45,46]
Postoperative pain	Inconsistent	Indirect and context-specific	Limited, procedure-specific RCT evidence	[46,47]
POD	Possible early reduction	Less RNB/hypoxia and lower anticholinergic burden	Small RCTs and large observational studies; mixed	[17,23,48,49,50,51]
Overall morbidity/mortality	Selected morbidity may improve	Reduction in selected complications, especially PPCs	Predominantly observational; indirect endpoints	[28,52,53]
QoR/LOS/cost	Mixed and domain-specific	Faster early recovery and fewer selected complications	Limited RCT and observational evidence; mixed	[18,28,54,55,56]

RNB, residual neuromuscular blockade; PPCs, postoperative pulmonary complications; PONV, postoperative nausea and vomiting; POUR, postoperative urinary retention; CRBD, catheter-related bladder discomfort; POD, postoperative delirium; QoR, quality of recovery; LOS, length of stay. Evidence strength reflects the consistency, size, and design of available studies rather than a formal GRADE assessment.

## 6. Safety Considerations and Special Populations

A balanced assessment of sugammadex must consider uncommon but potentially serious adverse effects. Marked bradycardia and rare cardiac arrest have been reported within minutes of administration, sometimes without other features of hypersensitivity [32]. Although comparative trials may show less frequent bradycardia than with neostigmine-based reversal, this does not negate the need for immediate hemodynamic monitoring and prompt treatment when clinically significant bradycardia occurs.

Hypersensitivity reactions range from isolated cutaneous manifestations to anaphylaxis and anaphylactic shock. In a clinical development study, hypersensitivity showed a dose-related pattern, and observational estimates indicate that anaphylaxis is rare but clinically consequential [57,58,59]. Most published reactions occur within the first few minutes after administration [57]. Clinicians should therefore maintain vigilance during emergence and the early post-administration period and treat suspected anaphylaxis according to established perioperative protocols. The available data do not support routine preoperative screening in patients without a relevant history.

Sugammadex can transiently prolong conventional coagulation assays. A randomized trial found limited increases in prothrombin time and activated partial thromboplastin time for less than 1 h without an associated increase in adjudicated bleeding, transfusion, or postoperative anemia [60]. A recent meta-analysis of randomized trials similarly found a small prolongation of prothrombin time without a consistent effect on activated partial thromboplastin time or established evidence of clinically important bleeding [61]. The clinical relevance therefore appears limited at routine doses in most patients, but caution and appropriate monitoring are reasonable in patients with coagulopathy, therapeutic anticoagulation, or a high bleeding risk, particularly when larger doses are used.

Residual or recurrent neuromuscular block can still occur after sugammadex, particularly when dosing is not matched to blockade depth, when neuromuscular monitoring is absent, or when redistribution of unbound NMBA exceeds the binding capacity of the administered dose. Quantitative monitoring should therefore be continued until sustained recovery is confirmed, rather than assuming successful reversal from drug administration alone [2,9,19,20].

Sugammadex and the sugammadex-NMBA complex are predominantly eliminated by the kidneys. Severe renal impairment markedly prolongs exposure, and current product information does not recommend sugammadex when creatinine clearance is <30 mL/min, including in patients receiving dialysis [62]. Systematic reviews and observational reports describe successful reversal in this population, but recovery may be slower and evidence concerning delayed recurarization and long-term safety remains limited [63,64]. If sugammadex is used despite severe renal impairment because the anticipated benefit is judged to outweigh the uncertainty, quantitative monitoring and extended postoperative surveillance are particularly important.

## 7. Concluding Remarks

In conclusion, sugammadex provides rapid and predictable reversal of aminosteroidal neuromuscular blockade when the dose is matched to measured blockade depth and consistently reduces RNB compared with neostigmine-based reversal. Associations with lower rates of selected PPCs, POUR, early PONV, and early physiological recovery have been reported, but effect estimates vary across outcomes, study designs, and clinical settings. Important safety considerations include rare bradycardia and anaphylaxis, transient coagulation assay changes, recurrent block after inadequate dosing, and prolonged exposure in severe renal impairment.

Evidence for improvement in global morbidity, mortality, postoperative pain, delirium, and patient-reported quality of recovery remains inconclusive. In particular, observational associations should not be interpreted as proof that sugammadex itself causes better downstream outcomes. The clinical effect of reversal is embedded within a broader management pathway that includes NMBA selection and cumulative dose, quantitative monitoring, depth-appropriate reversal, confirmation of sustained recovery, extubation practice, and postoperative care. Sugammadex should therefore be viewed as a useful component of optimized neuromuscular management rather than an independent determinant of postoperative recovery.

## Figures and Tables

**Table 1:** Pharmacologic and mechanistic differences between sugammadex and neostigmine-based reversal and their clinical implications.

	Sugammadex	Neostigmine-Based Reversal	Clinical Implication
Mechanism	Encapsulation of rocuronium/vecuronium	Acetylcholinesterase inhibition	Direct vs. indirect reversal
Effective depth	Deep, moderate, and shallow block	Mainly minimal to shallow block	Incomplete reversal risk differs by blockade depth
Need for anticholinergic	No	Yes	PONV, bradycardia, POUR, and CRBD implications
Autonomic effects	Minimal cholinergic effect	Muscarinic and antimuscarinic effects	Hemodynamic and GI/GU outcomes

Note: Quantitative neuromuscular monitoring is essential with either reversal strategy to determine blockade depth, guide depth-appropriate dosing, and confirm recovery before extubation. PONV, postoperative nausea and vomiting; POUR, postoperative urinary retention; CRBD, catheter-related bladder discomfort; GI, gastrointestinal; GU, genitourinary.

## Data Availability

No new data were created or analyzed in this study. Data sharing is not applicable to this article.

## Abbreviations

The following abbreviations are used in this manuscript:

AMG	acceleromyography
CRBD	catheter-related bladder discomfort
ECG	electrocardiography
EMG	electromyography
ENT	ear–nose–throat
GI	gastrointestinal
GU	genitourinary
LOS	length of stay
MACE	major adverse cardiovascular event
NMBA	neuromuscular blocking agent
NRS	numerical rating scale
PACU	post-anesthesia care unit
POD	postoperative delirium
PONV	postoperative nausea and vomiting
POUR	postoperative urinary retention
PPC	postoperative pulmonary complication
QoR	quality of recovery
RNB	residual neuromuscular blockade
TOF	train-of-four
VAS	visual analogue scale
